# Dietary Patterns and Their Relationship with Frailty in Functionally Independent Older Adults

**DOI:** 10.3390/nu10040406

**Published:** 2018-03-24

**Authors:** Mónica Machón, Maider Mateo-Abad, Kalliopi Vrotsou, Xabier Zupiria, Carolina Güell, Leonor Rico, Itziar Vergara

**Affiliations:** 1 Unidad de Investigación de Atención Primaria-OSIs Gipuzkoa, Osakidetza, 20014 San Sebastián, Spain; maider.mateoabad@osakidetza.eus (M.M.-A.); kalliopi.vrotsoukanari@osakidetza.eus (K.V.); CAROLINA.GUELLPELAYO@osakidetza.eus (C.G.); lricosanchez@hotmail.com (L.R.); itziar.vergaramitxeltorena@osakidetza.eus (I.V.); 2 Red de Investigación en Servicios de Salud en Enfermedades Crónicas (REDISSEC), 48902 Barakaldo, Spain; 3 Instituto de Investigación Sanitaria Biodonostia, 20014 San Sebastián, Spain; 4 Kronikgune- Centro de Investigación en Cronicidad, 48902 Barakaldo, Spain; 5 Centro de salud de Beraun, Osakidetza, 20100 Rentería, Spain; XABIER.XABIERZUPIRIA@osakidetza.eus; 6 Centro de salud de Alza, Osakidetza, 20017 San Sebastián, Spain

**Keywords:** nutrition, diet, frailty, older adults

## Abstract

The impact of dietary patterns rather than single foods or nutrients on health outcomes is increasingly recognized. This cross-sectional study examines the dietary patterns of 527 non-institutionalized functionally independent older people aged ≥70 years from Gipuzkoa (Spain). Sociodemographic characteristics, health status, anthropometric measures and dietary data are collected. Multiple correspondence analysis (MCA) and cluster analysis are performed to identify dietary patterns and groups of individuals. Frequency of selected food items and compliance with food recommendations are included in the MCA. A high proportion of the sample population are overweight or obese, whereas only 3.3% are at risk of malnutrition (determined with the Mini Nutritional Assessment). Frail individuals (*n* = 130), measured with the Timed-Up and Go test are older, have a lower educational level, are more obese, present a poorer health status (more depressive symptoms, polypharmacy and falls, among others) and worse compliance with food recommendations than robust individuals (*n* = 392). Three groups of individuals are identified: cluster one (*n* = 285), cluster two (*n* = 194) and cluster three (*n* = 48). A gradient of increasing frailty and poorer health status is observed from cluster one to cluster three. The latter also shows the poorest dietary pattern, regarding dietary recommendations. The use of an easy-to-use tool to assess diet allows detection of differences among the three clusters. There is a need to increase awareness on the implementation of nutritional screening and a subsequent dietary assessment in primary care settings to provide nutritional care to elder, and moreover, frail individuals.

## 1. Introduction

The aging of the population constitutes an important health challenge due to the increase in the life expectancy. Aging is a complex and multifactorial process accompanied by a series of physiological changes that lead to a progressive loss of adaptation to environmental demands and increasing vulnerability. One of the most severe expressions of aging is frailty [1]. Frailty is characterized by a progressive loss of functional capacity that can lead to dependence, institutionalization and death [2,3]. Unlike dependence, frailty may be reversible, and the effectiveness of interventions based on exercise, dietary improvement or control of polypharmacy is well known [4,5].

Diet is one of the pillars of health. Unhealthy diet constitutes an important risk factor for chronic pathologies, such as diabetes, cancer or cardiovascular diseases [6]. An inappropriate nutritional status increases the risk of frailty, in older people. More specifically, an insufficient consumption of protein and micronutrients like vitamin D, C and omega-3 fatty acids are associated with this condition [7,8,9]. Quite the reverse, the adherence to healthy dietary patterns, such as the Mediterranean diet, might be able to prevent it [8]. This dietary pattern is mainly characterized by an abundant consumption of plant food, with olive oil as a principal source of fat, high to moderate amounts of fish, moderate intake of eggs, poultry and dairy products and low consumption of red meat [10].

The assessment of dietary characteristics in older people constitutes a challenge. Aging is associated with short term memory loss that can alter their ability to remember all the food and drinks consumed and, consequently, underreporting of intake can be observed. Food frequency questionnaires, 24-hour dietary recall or 7-day food records [11] are some of the methods used for collecting food consumption data in such populations. However, to date, none of them have been incorporated into routine practice in primary care settings, mainly due to the amount of time required for their administration. The assessment of food intake, especially in the frail, is a key element in identifying which food groups are consumed inadequately. This knowledge is valuable to maintain an appropriate nutritional status [12] and to battle the condition of frailty itself.

The objective of this study is to examine the dietary patterns in a sample of non-institutionalized functionally independent older people living in the province of Gipuzkoa (Basque Country, Spain) and to explore potential associations between these patterns and frailty.

## 2. Material and Methods

### 2.1. Design

This is a one-year follow-up cohort study; intending to explore, among others, the relationship between frailty status and adverse events at the end of the follow-up. The data reported here are exclusively based on the baseline assessment of the cohort.

### 2.2. Study Population

Non-institutionalized individuals aged 70 years or older, with a Barthel index score [13,14] higher than 90 points who agreed to participate are included in the study. Terminally ill subjects or those residing more than six months in a different area are excluded.

### 2.3. Data Collection

Individuals were first randomly selected from administrative databases of four primary care centers (PCC) of Gipuzkoa (i.e., Urnieta, Getaria, Zumaia and Zestoa). Those with a registered Barthel lower than 60 in their clinical health records, were excluded. All initially included individuals were invited to take part in the study. They were contacted first by letter and then by phone and their willingness to participate was recorded. Three trained nurses, one of whom also had a human nutrition university degree, oversaw the field work and performed the baseline assessment in the corresponding PCC. Given that the purpose of the current work was to study frail, not dependent individuals, only autonomous subjects (Barthel > 90 points) were considered.

All participants signed an informed consent. A blood sample was obtained from those who gave authorization to study biomarkers of frailty. The data collection was conducted between August 2016 and October 2017. The study received authorization from the Euskadi Ethics Committee (CEIC Euskadi 11/2015).

### 2.4. Variables

The following variables were obtained: sociodemographic characteristics, health status, anthropometric measurements and diet assessment.

### 2.5. Frailty and Health Status 

Frailty was evaluated with the Timed Up and Go test (TUG). The TUG [15] measures the time that an adult needs to get up from a chair, walk three meters, turn around, come back to the chair and sit down. The person is considered to be frail if the time to perform the test is longer than 12 seconds. Self-perceived health was explored through a single item (“Overall, you would say that your health is…”) for which five response options were grouped in two categories: good (very good/good) and poor (fair/poor/very poor). Presence of depressive symptoms was considered if the score obtained in the Geriatric Depression Scale was 5 or more [16,17]. Polypharmacy was defined as the consumption of 4 or more prescription drugs per day [18]. Participants also reported sight and hearing difficulties and if they have had fractures or had fallen during the last 12 months.

### 2.6. Anthropometric Measures and Diet Assessment

Body mass index (BMI) was calculated as weight over height squared. The BMI was classified as: low normal weight (<23 kg/m^2^), normal weight (23–24.9 kg/m^2^), overweight (25–29.9 kg/m^2^) and obese (≥30 kg/m^2^) [19]. The single underweight subject (BMI <18.5 kg/m^2^) was included in the low normal weight category. Waist-to-hip-ratio (WHR) was estimated by dividing waist circumference by hip circumference. Abdominal obesity was defined as WHR ≥ 0.90 for males and ≥0.85 for females [20]. Risk of malnutrition was studied with the Mini Nutritional Assessment scale (MNA) [21]. Participants were categorized as: normal nutrition (score 24–30), risk of malnutrition (score 17–23.5) and malnourished (score < 17). Frequency of food consumption was studied by using an extended version of a questionnaire included in the Basque Health survey [22]. Participants were asked how many times per week they ate: fresh fruit; natural juice; vegetables; legumes; pasta, rice and potatoes; bread and cereals; milk, cheese and yogurt; eggs; red meat; white meat; lean fish; fatty fish; cold cured meat; raw nuts; soft drinks; salty snacks; sweets; and fast food. Response options were: daily, ≥3 times/week, 1–2 times/week, <1 time/week, and never or almost never. For “daily” responses, the number of portions was recorded not only for fruit, juices and vegetables [22] but also for meat, fish, pasta-rice-potatoes, bread-cereals, legumes, milk-cheese-yogurt. Additionally, the number of meals, glasses of water and tablespoons of olive oil per day was registered. Different portion sizes were used (e.g., a handful for raw nuts, a tablespoon for olive oil, a unit for yogurt and eggs, one meat steak and one fish steak). The foods added by the current research group for creating the extended version of the consumption questionnaire were: raw nuts, olive oil, number of meals and glasses of water; the type of meat and fish, type of dairy products and type of bread-cereals consumed. The above information was also used to study if the participants complied with the food recommendations of the healthy eating pyramid of the Spanish Society of Community Nutrition (SENC) [23], based on the Mediterranean diet. The following SENC daily consumption recommendations have been considered: three or more servings or pieces of fruit; at least two servings of vegetables; at least two tablespoons of olive oil; 2–3 servings of milk and dairy products; three or more portions of cereals (bread is also included); four or more glasses of water; eating fish, white meat, eggs, nuts or legumes ≥ 1 time per day; and finally, red meat ≤ 1–2 times per week.

### 2.7. Statistical Analysis

Categorical variables were described as frequencies and percentages (%), and continuous variables as mean with standard deviations (SD). A Chi-squared test and Student´s *t*-test or the non-parametric Wilcoxon test were used to compare categorical and continuous variables, respectively.

A combination of multiple correspondence analysis (MCA) and cluster analysis were used to summarize the information recorded by several diet variables to obtain dietary patterns and then to differentiate groups of individuals with respect to those patterns. Both are multivariate techniques widely utilized in medical research [24,25]. First, an MCA was performed on all individuals to identify dietary patterns, including in the analysis the categorical variables related to diet, such as compliance with SENC recommendations and food intake. This approach allows to summarize the information of various categorical variables into a few dimensions, which explain the maximum amount of variability contained in the active variables included in the analysis [24]. The following active variables were introduced in the MCA in this case: frequency of natural juice, red and white meat, lean and fatty fish, pasta-rice-potatoes, eggs, legumes, cold cured meat, raw nuts, soft drinks, fast food, salty snacks and sweets. Compliance with the SENC food recommendations per variable (yes/no) was included for fresh fruit, vegetables, olive oil, milk-cheese-yogurt, bread-cereals and water, which are usually consumed on a daily basis. Categories for red and white meat, lean and fatty fish, soft drinks and fast food were grouped due to low frequency responses obtained (frequency < 10). This was done to avoid low frequency category values reaching high representativeness in the MCA results. Additionally, sex, TUG and MNA were considered as illustrative variables which were not included in the MCA analysis, but were added to the results to check their association with the active variables. MCA results can be graphically represented on a map, showing each category of the variables or each individual as a point, plotted in the dimensions constructed by the MCA. The closer the points the stronger the relationship between the categories/individuals. Second, a hierarchical cluster analysis was applied based on the dimensions provided by the MCA to organize all participants into groups of similar individuals regarding dietary patterns. The clusters obtained were displayed in the map constructed by the MCA dimensions. To describe and characterize the clusters, a comparison between groups was performed using several sociodemographic characteristics, health status, anthropometric and nutritional and diet variables, not only those included in the MCA analysis. Statistical analysis was carried out using the free statistical software R (version 3.4.0, R Foundation for Statistical Computing, Vienna, Austria).

## 3. Results

A total of 527 individuals were included in the study, 55% being women and with an overall mean age of 76.22 (SD 5.21) years (Table 1). Participants had a good perceived health (77.4%) even though the presence of polypharmacy was high (43.4%) as well as sight (75.8%) and hearing (40.2%) impairments. The prevalence of frailty, measured with the TUG, was 24.9%. Overweight and obesity were highly prevalent in the studied sample (45.5% and 31%, respectively). The proportion of individuals at risk of malnutrition was very low (3.3%). A high percentage of the sample complied with the recommendations related to olive oil, cereals, fish-white meat-eggs-nuts-legumes and red meat.

**Figure 1:**
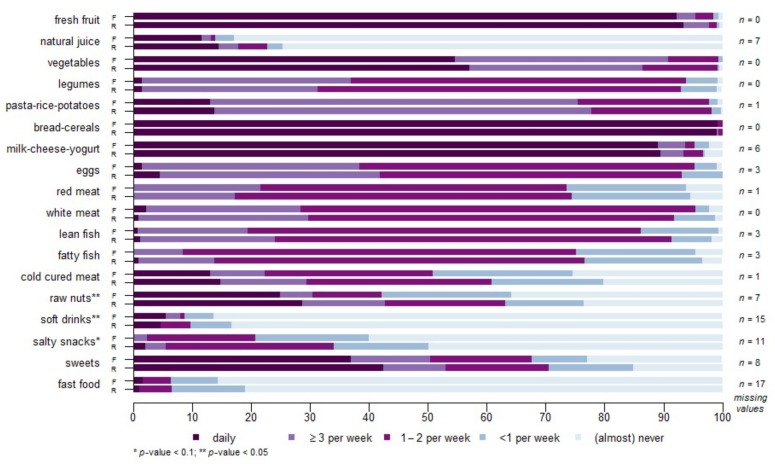
Frequency of food intake in frail (F) and robust (R) individuals. Statistically significant differences (*p* < 0.05) are labeled with a double asterisk. The numbers given on the right side of the graph correspond to missing values.

Relevant significant differences were observed among robust and frail individuals. Frail individuals were significantly older and had a lower educational level. They were also more likely to report poor health, depressive symptoms, polypharmacy and falls. Regarding nutritional status, frail participants showed more obesity and low normal weight, with higher values of waist and hip circumferences and were much more likely to present risk of malnutrition. When diet was assessed, certain significant differences were observed between these groups. Specifically, frail individuals consumed less raw nuts and drank more soft drinks (Figure 1). Differences were also observed in the compliance with the SENC food recommendations. Only vegetable consumption was statistically significant, lower in the frail group. Nonetheless, robust participants complied better in all remaining food recommendations, even though observed differences did not reach statistical significance.

**Figure 2:**
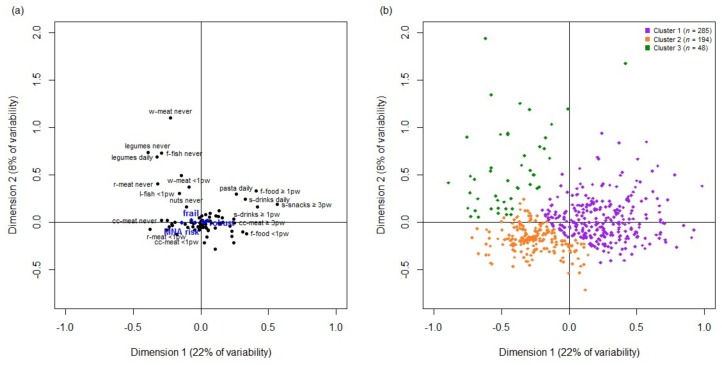
Graphical display of the first two dimensions of the multiple correspondence analysis (MCA) (**a**) and cluster analysis of individuals (**b**). (**a**) has black dots in the plane representing the categories of the active diet variables included in the MCA, only the most representative ones were labeled. Blue dots show the relative position of illustrative variables (frailty status, Mini-Nutritional Assessment-MNA and sex). The closer the points are, the stronger the relationship between the categories. Abbreviations: f-fish, fatty fish; r-meat, red meat; cc-meat, cold cured meat; f-food, fast food; s-snacks, salty snacks; s-drinks, soft drinks; pw, per week; (**b**) has relative position of the individuals represented by different colors, depending on the subtype provided by the cluster analysis.

The MCA results are shown in Figure 2. Two dimensions explained 21.7% and 8.0% of the variability in the data, respectively. The figure showed how the diet information is summarized in the two dimensions obtained with the MCA. The amount of food consumed is low on the left side and normal-high on the right side in the first dimension. The second dimension highlights the low frequency intake of certain foods (e.g., white and red meat, fatty fish or raw nuts) observed on the positive side of that dimension. The cluster analysis considering the two dimensions of the MCA identified three groups of individuals. Those pertaining to cluster 1 (*n* = 285) are located on the positive side of dimension 1. Cluster 2 (*n* = 194) and cluster 3 (*n* = 48) are situated on the negative side of dimension 1 and differentiated by dimension 2.

**Figure 3:**
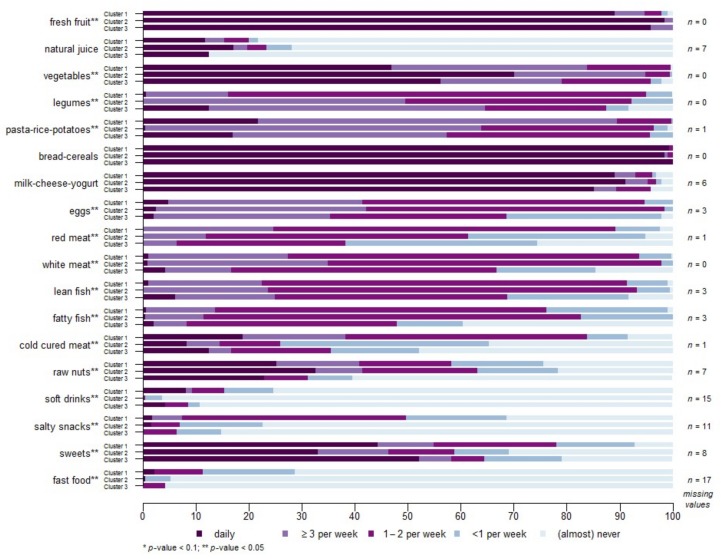
Frequency of food intake in the three clusters of individuals identified. All *p*-values are statistically significant (*p* < 0.05) except for natural juice (*p* = 0.109), bread-cereals (*p* = 0.682) and milk-cheese-yogurt (*p* = 0.775). The number given on the right side of the graph corresponds to missing values.

Table 2 and Figure 3 include a characterization of the three clusters and a comparison between them. Cluster 1 was constituted by patients with a mean age of 76.31 (SD 5.18) years and a frailty prevalence of 20.7%. Compared to the other two clusters, this group of individuals was more balanced by sex (48.8% were women), showed a lower frequency of depressive symptoms (10.7%), polypharmacy, sight problems and antecedents of falls (not statistically significant differences). They also ate less meals per day. Regarding the SENC indications, this group complied with cereals intake but less with fruit and red meat recommendations.

Individuals pertaining to cluster 2 were younger and 63.1% were women. The prevalence of frailty in this group was 28.1%, higher than cluster 1 but lower than cluster 3. These people were less likely to have hearing limitations (32.1%) compared to the other two clusters. This cluster was characterized by a high compliance for fruit and milk-dairy products, and moderate compliance of meat recommendations, while they also ate more meals per day.

The third cluster gathered the oldest individuals, with more women (66.7%) and the lowest educational level, while one of every three subjects of this group were living alone. It also had the highest prevalence of frailty (37.8%) and the worst health status, given that 25% of the respective individuals reported a poor self-perceived health. Simultaneously, cluster 3 individuals presented the highest prevalence of: depressive symptoms, polypharmacy, fractures and falls. Regarding nutritional status, this group had more obesity and low normal weight and the highest risk of malnutrition. Finally, this cluster showed the lowest level of compliance with the SENC recommendation for vegetables, cereal, milk-dairy products and fish-white meat-eggs-nuts-legumes intake, and the highest regarding red meat intake. Even though some of the above mentioned differences did not reach statistical significance on an individual level, as a whole, cluster 3 presented worse health and dietary patterns according to the SENC recommendations.

## 4. Discussion

Dietary patterns and their potential associations with frailty is examined in the present work. Although the studied sample presented certain health problems, reflected in polypharmacy, sight and hearing limitations, the majority perceived their health as being good. The prevalence of frailty observed in the data was similar to other studies included in the systematic review of Collard et al. [26]. A low proportion of the individuals had normal weight and were at risk of malnutrition. Moreover, a high compliance with the SENC food recommendations for olive oil, cereals, fish-white meat-eggs-nuts-legumes and read meat was observed. Similarly, in a Spanish study of older people [27], a high percentage of the sample complied with the Mediterranean Diet recommendations for cereals, olive oil and, fruit, milk and dairy products. Although, it is important to note that the recommendations used in that study were less demanding than those included in this work. The daily consumption of fruit, vegetables, pasta-rice-potatoes, bread and milk-cheese-yogurt was higher in this sample compared to the Basque Health Survey [22], lower for legumes and similar for eggs.

When comparing robust and frail individuals, several differences emerged. Frail individuals were older, had a lower educational level and a worse health status. These facts are already known and consistent with the literature [28,29]. They also showed more obesity and low normal weight and a worse compliance with the SENC recommendations. Previous evidence exists that diet is an important factor closely related to frailty [8] and these findings seem aligned with this association. Despite the fact that statistically significant differences were not seen in all food related variables, worse overall diet patterns corresponded to clusters with a higher prevalence of frailty. Additionally, it is relevant to mention the difficulty in comparing the current diet assessment results with other studies due to different applied methodologies [29,30,31].

Previous studies have explored dietary patterns in aged populations by using factor analysis [28,32], principal component analysis [33] or factor analysis combined with cluster methods [34]. Those studies featuring dietary patterns were assessed without a posterior differentiation of groups of individuals in relation to these patterns [28,33]. In another work, only the characteristics of the non-frail participants according to different dietary patterns [32] were presented. The combination of the two multivariate techniques that were applied in the current work constitutes a more comprehensive methodological approach.

Three groups of individuals emerged through the combined used of MCA and cluster analysis techniques. Beginning with cluster 1 through to cluster 3, a gradient of increasing frailty and worse health status (polypharmacy, depression and fractures) was observed, with the last cluster presenting the highest mean age and proportion of lower educated women, more frailty, poor self-perceived health and polypharmacy, among others.

Regarding nutritional assessment, cluster 3 also had the highest percentage of low normal weight and obese individuals and the highest risk of malnourishment. Interesting results were observed when exploring the dietary patterns of the three clusters. Cluster 1 had a low compliance with vegetable and fruit recommendations and the lowest with red meat indication. Conversely, this cluster had a medium compliance with dairy product recommendations and the highest with cereals, fish and white meat. The above reflects a pattern based on high meat and cereals intake, medium dairy products and low fruit and vegetables consumption. Regarding cluster 2, the most relevant characteristics of their dietary pattern is the high compliance with fruit, vegetables, olive oil, milk and dairy products, fish and meat, both white and red. This group presented an overall high adherence to the SENC recommendations. Finally, cluster 3 presented the lowest level of compliance with the SENC recommendations, except for red meat consumption.

Where dietary patterns are concerned, certain particularities of this study should be considered. The authors are aware that complex nutritional component assessment is the gold standard for describing dietary patterns and identifying nutritional deficits in the population of interest, but the authors are also aware that these kinds of tools have not been designed for clinical practice. The tools´ extension and administration time is an obstacle in their implementation in clinical setting, let alone during primary care consultations. Therefore, for the current work and considering its further transferability to primary care settings, the authors opted for a simple nutritional assessment tool. The Basque Health Survey questionnaire [22], which was subsequently expanded, allowed for the measurement of key diet components and more specific amounts. This approach far from being a limitation constitutes a strength of the current work. The tool, despite its constraints, managed to identify different dietary patterns in subjects of different frailty levels.

While this is a cross sectional study and no causal relationships can be established, an association seems to exist between a poor diet with bad health status and frailty. Individuals with a great disease load, bad health perception and a high risk of becoming dependent and institutionalized (cluster 3) also presented a very poor diet pattern that is known to increase the aforementioned risks. The causes of poor diet in aged and sick populations are well known and have been described—poor dentition, alteration of taste and smell, loss of appetite, swallowing disorders or functional difficulties, which complicate meal preparation [35,36]. However, the assessment of some of these problems, and the way the latter may affect dietary patterns, has not been systematically included in the care plans provided to elder populations in primary care settings.

Strengths of this study deserve to be highlighted. First, a big sample of functionally independent community dwelling individuals was studied. Another relevant aspect was exploring the data by applying MCA and cluster analysis. Thanks to the use of both techniques, it was possible to identify and characterize different groups of individuals according to their health status and dietary habits. Finally, the simple assessment tools used and the promising findings may serve as a basis for further investigative opportunities in primary care settings, and facilitate the implementation of future strategies.

Conversely, certain limitations also should be mentioned. Although the food consumption questionnaire included in the study was not as extensive compared to other works [28], some recall bias may have affected in the results. Previous studies also have described this situation [28,32,37]. Missing information observed in questions related to the frequency of food consumed was not substantial, in this sense. However, when the number of portions was asked of individuals consuming foods “daily”, the proportion of missing data increased, especially for olive oil, vegetables, fresh fruit and dairy products. It seemed that older people could not easily remember such information, or they found it difficult to quantify the amounts consumed.

The identification of different dietary patterns is a key element to characterize and tackle the health needs of aging populations. This is especially relevant in the case of frail patients. Further research and prospective studies are needed to better understand the impact of nutritional status and dietary patterns on the development of frailty. An appropriate dietary assessment adequate to be used in primary care settings is of major importance. It can help tackle problems stemming from inadequate dietary habits and promote healthy dietary patterns, such as the Mediterranean diet [10].

## 5. Conclusions

This study of functionally independent community dwelling older adults had three different groups of individuals derived according to food consumption. A gradient of increasing frailty, poorer health status and worse dietary pattern, regarding recommendations, was observed in the studied sample. The results obtained showed that even with the utilization of a simple tool to assess diet, differences between the dietary patterns of the three clusters were found. Therefore, it is necessary to raise awareness among primary health care professionals in relation to the importance of the implementation of nutrition screening tools, such as MNA, and a subsequent dietary assessment not only if risk of malnutrition or malnutrition is detected but also for further evaluation of frail patients´ dietary habits. Three key elements should be considered when deploying assessment procedures in primary care settings: adequate assessment tools, training for primary care teams and expert professionals for referrals.

## Figures and Tables

**Table 1:** Characteristics of the sample according to frailty status, measured with Timed-Up-and-Go test.

Variables	Total (*n* = 527)	Missing	Frail (*n* = 130)	Robust (*n* = 392)	*p*-Value
Age in years, mean (SD)	76.22 (5.21)	0	78.46 (5.52)	75.43 (4.82)	<0.001
Sex		0			0.262
Men	237 (45%)		53 (40.8%)	184 (46.9%)	
Women	290 (55%)		77 (59.2%)	208 (53.1%)	
Educational level		0			0.002
Primary or lower	415 (78.7%)		116 (89.2%)	294 (75%)	
Secondary	25 (4.7%)		2 (1.5%)	23 (5.9%)	
Vocational training or university	87 (16.5%)		12 (9.2%)	75 (19.1%)	
Living alone	121 (23%)	0	25 (19.2%)	93 (23.7%)	0.347
Self-perceived health		1			0.005
Good	407 (77.4%)		88 (68.2%)	316 (80.6%)	
Poor	119 (22.6%)		41 (31.8%)	76 (19.4%)	
Geriatric Depression Scale		54			<0.001
Depressive symptoms (≥5)	66 (14%)		30 (27.8%)	35 (9.6%)	
Not depressive symptoms (<5)	407 (86%)		78 (72.2%)	328 (90.4%)	
Polypharmacy (≥4 drugs)	226 (43.4%)	6	77 (59.7%)	146 (37.6%)	<0.001
Sight impairments	398 (75.8%)	2	102 (78.5%)	291 (74.6%)	0.444
Hearing limitations	211 (40.2%)	2	60 (46.2%)	148 (37.9%)	0.121
Falls during the last year	124 (23.8%)	7	43 (33.1%)	78 (20.3%)	0.004
Fractures during the last year	30 (5.8%)	8	12 (9.3%)	17 (4.4%)	0.063
Height, mean (SD) (cm)	161.11 (9.25)	0	159.08 (9.07)	161.86 (9.23)	0.003
Weight, mean (SD) (kg)	73.35 (13.47)	0	73.84 (14.41)	73.2 (13.2)	0.656
Body Mass Index		0			0.001
Low normal weight (<23 kg/m^2^)	50 (9.5%)		9 (6.9%)	41 (10.5%)	
Normal weight (23–24.9 kg/m^2^)	73 (13.9%)		17 (13.1%)	56 (14.3%)	
Overweight (25–29.9 kg/m^2^)	240 (45.5%)		46 (35.4%)	191 (48.7%)	
Obesity (≥30 kg/m^2^)	164 (48%)		58 (44.6%)	104 (26.5%)	
Waist circumference, mean (SD) (cm)	95.04 (12.5)	0	97.06 (13.06)	94.31 (12.23)	0.036
Hip circumference, mean (SD) (cm)	104.04 (8.7)	0	105.57 (10.06)	103.48 (8.14)	0.033
Waist-to-hip-ratio		0			0.262
Normal	160 (30.4%)		34 (26.2%)	125 (31.9%)	
High	367 (69.6%)		96 (73.8%)	267 (68.1%)	
Mini Nutritional Assessment		17			<0.001
Normal (24–30)	493 (96.7%)		114 (90.5%)	375 (98.7%)	
At risk of malnutrition (17–23.5)	17 (3.3%)		12 (9.5%)	5 (1.3%)	
Malnourished (<17)	0 (0%)		0 (0%)	0 (0%)	
Daily consumption					
Number of meals, mean (SD)	3.7 (0.73)	8	3.7 (0.69)	3.69 (0.73)	0.954
Number of glasses of water, mean (SD)	3.75 (1.86)	22	3.75 (1.72)	3.74 (1.90)	0.977
Number of tablespoons of olive oil, mean (SD)	2.6 (0.89)	65	2.52 (0.84)	2.62 (0.91)	0.317
Portions of fresh fruit, mean (SD)	2.84 (1.18)	36	2.72 (1.06)	2.89 (1.22)	0.141
Portions of vegetables, mean (SD)	1.22 (0.43)	237	1.1 (0.30)	1.26 (0.46)	0.001
Portions of milk-cheese-yogurt, mean (SD)	2.35 (1.01)	64	2.45 (1.07)	2.32 (1.00)	0.258
Meet daily recommendation					
Fresh fruit (≥3 portions)	282 (53.5%)	0	67 (51.5%)	214 (54.6%)	0.615
Vegetable (≥2 portions)	62 (11.9%)	7	7 (5.5%)	55 (14.2%)	0.013
Olive oil (≥2 tablespoon)	420 (90.9%)	65	96 (89.7%)	320 (91.2%)	0.792
Milk and dairy products recommendation (2–3 portions)	309 (60%)	12	72 (56.7%)	232 (60.6%)	0.504
Cereals recommendation (≥3 portions)	387 (76.3%)	20	91 (71.7%)	295 (78.5%)	0.148
Water recommendations (≥4 glasses)	265 (52.5%)	22	65 (52.8%)	197 (52.3%)	0.992
Fish-white meat-eggs-nuts-legumes (any ≥1 portions)	465 (88.2%)	0	113 (86.9%)	349 (89%)	0.621
Meet weekly recommendation					
Red meat (≤1–2 portions)	430 (87.7%)	1	101 (78.3%)	324 (82.7%)	0.329

**Table 2:** Characterization of the clusters of individuals and comparison between clusters.

Variables	Cluster 1 (*n* = 285)	Cluster 2 (*n* = 194)	Cluster 3 (*n* = 48)	*p*-Value
Age, mean (SD)	76.31 (5.18)	75.6 (4.84)	78.19 (6.29)	0.008
Sex				0.006
Men	146 (51.2%)	75 (38.7%)	16 (33.3%)	
Women	139 (48.8%)	119 (61.3%)	32 (66.7%)	
Educational level				0.098
Primary or lower	213 (74.7%)	159 (82%)	43 (89.6%)	
Secondary	16 (5.6%)	7 (3.6%)	2 (4.2%)	
Vocational training or university	56 (19.6%)	28 (14.4%)	3 (6.2%)	
Living alone	69 (24.2%)	38 (19.6%)	14 (29.2%)	0.280
Timed up and go				0.021
Frail (>12 s)	59 (20.7%)	54 (28.1%)	17 (37.8%)	
Robust (≤12 s)	226 (79.3%)	138 (71.9%)	28 (62.2%)	
Self-perceived health				0.469
Good	216 (75.8%)	155 (80.3%)	36 (75%)	
Poor	69 (24.2%)	38 (19.7%)	12 (25%)	
Geriatric Depression Scale				0.001
Depressive symptoms (≥5)	29 (10.7%)	24 (15%)	13 (31.7%)	
Not depressive symptoms (<5)	243 (89.3%)	136 (85%)	28 (68.3%)	
Polypharmacy (≥4 drugs)	115 (40.5%)	84 (44.2%)	27 (57.4%)	0.091
Sight impairments	213 (74.7%)	143 (74.5%)	42 (87.5%)	0.139
Hearing limitations	123 (43.3%)	62 (32.1%)	26 (54.2%)	0.006
Falls during the last year	66 (23.4%)	40 (21.1%)	18 (37.5%)	0.056
Fractures during the last year	11 (3.9%)	11 (5.8%)	8 (16.7%)	0.002
Height, mean (SD) (cm)	162.98 (9.46)	159.05 (8.58)	158.33 (8.24)	<0.001
Weight, mean (SD) (kg)	75.25 (14.27)	71.33 (12.25)	70.2 (11.59)	0.002
Body Mass Index				0.998
Low normal weight (<23 kg/m^2^)	26 (9.1%)	19 (9.8%)	5 (10.4%)	
Normal weight (23–24.9 kg/m^2^)	39 (13.7%)	28 (14.4%)	6 (12.5%)	
Overweight (25–29.9 kg/m^2^)	130 (45.6%)	89 (45.9%)	21 (43.7%)	
Obesity (≥30 kg/m^2^)	90 (31.6%)	58 (29.9%)	16 (33.3%)	
Waist circumference, mean (SD) (cm)	95.47 (12.53)	95.07 (12.82)	92.45 (10.77)	0.302
Hip circumference, mean (SD) (cm)	103.91 (8.48)	104.23 (9.26)	104 (7.68)	0.925
Waist-to-hip-ratio				0.506
Normal	83 (29.1%)	59 (30.4%)	18 (37.5%)	
High	202 (70.9%)	135 (69.6%)	30 (62.5%)	
Mini Nutritional Assessment				0.327
Normal (24–30)	275 (97.5%)	175 (96.2%)	43 (93.5%)	
At risk of malnutrition (17–23.5)	7 (2.5%)	7 (3.8%)	3 (6.5%)	
Malnourished (<17)	0	0	0	
Daily consumption				
Number of meals per day, mean (SD)	3.58 (0.69)	3.87 (0.75)	3.7 (0.75)	<0.001
Number of glasses of water, mean (SD)	3.61 (1.88)	4.01 (1.74)	3.54 (2.08)	0.055
Number of tablespoons of olive oil, mean (SD)	2.51 (0.89)	2.75 (0.90)	2.55 (0.80)	0.033
Portions of fresh fruit, mean (SD)	2.61 (1.19)	3.14 (1.07)	2.85 (1.28)	<0.001
Portions of vegetables, mean (SD)	1.25 (0.45)	1.21 (0.43)	1.15 (0.37)	0.558
Portions of milk-cheese-yogurt, mean (SD)	2.42 (1.12)	2.23 (0.82)	2.48 (1.04)	0.124
Meet daily recommendation				
Fresh fruit recommendation (≥3 portions)	116 (40.7%)	138 (71.1%)	28 (58.3%)	<0.001
Vegetable recommendation (≥2 portions)	31 (11%)	27 (14.1%)	4 (8.5%)	0.456
Olive oil recommendation (≥2 tablespoon)	238 (89.1%)	147 (93.6%)	35 (92.1%)	0.289
Milk and dairy product recommendation (2–3 portions)	152 (54.5%)	133 (70.4%)	24 (51.1%)	0.001
Cereals recommendation (≥3 portions)	244 (87.5%)	117 (63.9%)	26 (57.8%)	<0.001
Water recommendations (≥4 glasses)	126 (45.7%)	117 (63.9%)	22 (47.8%)	0.001
Fish-white meat-eggs-nuts-legumes (any ≥1 portions)	249 (87.4%)	175 (90.2%)	41 (85.4%)	0.522
Meet weekly recommendation				
Red meat (≤1–2 portions)	215 (75.4%)	171 (88.1%)	44 (93.6%)	<0.001
